# Combining Raman Microspectroscopy and X-ray Microcomputed Tomography for the Study of Bone Quality in Apolipoprotein-Deficient Animal Models

**DOI:** 10.3390/molecules28207196

**Published:** 2023-10-20

**Authors:** Stefani Fertaki, Panagiota Giannoutsou, Malvina G. Orkoula

**Affiliations:** 1 Department of Pharmacy, University of Patras, 265 04 Patras, Greece; 2 Foundation for Research and Technology, Institute of Chemical Engineering and High Temperatures, FORTH/ICE-HT, 265 04 Patras, Greece

**Keywords:** raman spectroscopy, bone quality, mice, osteoporosis, collagen crosslinks, microcomputed tomography

## Abstract

Raman microspectroscopy and X-ray microcomputed tomography (micro-CT) were used for assessment of the quality of the femur and tibia bones in apolipoprotein-deficient mice compared to control littermates. The cortical and trabecular bone was investigated separately. Raman spectra revealed no differences in the bioapatite-to-collagenous matrix ratio of the cortical bone. The quantities of calcium and collagen, which were measured using atomic absorption spectrometry and thermogravimetric analysis, respectively, were also found to be equal in the two groups. Density and morphometric parameters, which were measured using micro-CT, verified the cortical mineral stability. Bone quality indices were measured using Raman spectra. A decreased collagen crosslink (trivalent-to-divalent) ratio revealed delayed maturation of the collagen network. Such a decrease has been reported in the literature to be connected to decreased bone strength. For the trabecular bone, micro-CT revealed severe osteoporosis in the knock-out group, which was evident from a decreased mineral density, trabecular thickness and increased bone surface/volume ratio. The trabecular bone was not accessible for Raman spectroscopy. According to these results, the cortical and trabecular femur bone is expected to exhibit proneness to fracturing, each for a different reason. A combination of the two techniques was regarded as necessary for an overall assessment of bone quality.

## 1. Introduction

### 1.1. Bone and Its Constituents

Bone exists in two macroscopic forms in the skeleton: the strong and compact type found in the outer layers of long bones, which is called the cortical bone, and the spongy-looking porous inner type at the ends of long bones and vertebrae, which is called the trabecular or cancellous bone.

Bone is a two-phase material consisting of an inorganic carbonated calcium phosphate mineral (bioapatite) fraction and an organic component dominated by collagen. The quantity and quality of these main bone constituents are important for bone integrity. The term “bone quality” refers to an assembly of three major parameters: the composition, microstructure and mechanical properties of bone tissue. These are interrelated and together determine bone’s behavior as a material and its ability to perform mechanical functions (body movement, joint function and skeleton support) [1,2]. The mineral content of bone is a poorly crystalline carbonate and acidic phosphate-substituted nonstoichiometric analog of hydroxyapatite. The stiffness and brittleness of bone tissue are, at least in part, determined by its mineral content and composition [3]. It is widely reported that the mineral content of bone increases with age [4,5,6], reaching a maximum (at the age of 50 for humans) and then decreasing [7,8].

Collagen is the most abundant protein in the organic matrix of mineralizing tissues. Collagen networks serve the task of creating and keeping bone tough and strong. Toughness, which is necessary to resist fracture and reinforce strength, depends on a collection of covalent collagen crosslinks. One of the most critical properties of collagen is its crosslinking pattern. Divalent (immature) crosslinks (dihydroxylysinorleucine (DHLNL) and hydroxylysinorleucine (HLNL)) are formed by the reaction of lysyl-oxidase-produced allysines with helical hydroxylysines on neighboring collagen molecules. A fraction of these crosslinks will subsequently mature with hydroxylysyl aldehyde to trivalent forms, which are known to include the pyridinolines hydroxylysyl pyridinoline (HP) and lysyl pyridinoline (LP), as well as pyrroles. Their ratio reflects the collagen network’s maturity. Advanced glycation end-product (AGE) crosslinks, such as pentosidine (PEN), form independently of enzyme activity in the presence of sugars [9,10]. Shifts in the crosslink profile occur in many bone pathologies, as well as with age. Understanding which of these changes are significant to bone-fracture resistance and bone strength is critical [11,12,13,14].

The organic matrix of bone has received considerably less attention than its mineral content. It has been recorded that the matrix content is decreased in osteoporosis. Furthermore, the mature-to-immature crosslink ratio (CLR) is higher in osteoporotic bones compared to their age- and sex-matched controls. The CLR also changes over the life of bone tissue. Young tissue lacks mature pyridinoline crosslinks. As the tissue matures, the trivalent-to-divalent crosslink ratio increases [13,14]. Toughness is supplied primarily by the organic matrix. The task of stabilizing the polymeric network depends on a collection of covalent crosslinks [1,15].

### 1.2. Bone and Raman Spectroscopy

Raman spectroscopy is a chemical-analysis technique that has been extensively used for the characterization and quantification of bone constituents [1,2,15]. The main advantages of Raman spectroscopy in the analysis of bone include its applicability to fresh tissue and its high spatial resolution (with sampling volumes of 1 μm^3^ or less) [2].

Bone Raman metrics, calculated as band-intensity ratios in the Raman spectra of bone, correspond to the quantity ratio of mineral to collagen and collagen matrix evaluation. They have been reported in the literature to depend on bone tissue quality and age, and their changes are related to bone pathological situations. Mineral quality metrics include the mineral-to-matrix ratio, the substitution of phosphate ions with carbonates and apatite particle crystallite size distribution. These are also related to bone age and health. The mineral-to-matrix ratio was reduced in the cancellous bone of the lumbar spine in osteoporotic humans [16]. An increased carbonate substitution of phosphates has been reported in the femora of women who have sustained a fracture compared to their unfractured controls [17]. Tibiae from mice with rheumatoid arthritis exhibited a reduced mineral-to-matrix ratio and an increased carbonate-to-phosphate ratio [18]. The stiffness and bending modulus of bone were found to be significantly dependent on the degree of mineralization, crystallinity and carbonate-to-phosphate ratio [19]. The mineral-to-matrix ratio and carbonate-to-phosphate ratio were thought, until recently, to be the strongest predictors of mechanical properties [15].

### 1.3. Bone and Microcomputed Tomography

Microcomputed tomography is an imaging technique that can be used to quantitatively represent bone geometry (quantitative morphometry). Bone mineral density (following calibration of values with a tissue “phantom” of a known mineral density, serving as a standard), trabecular thickness, trabecular separation, thickness distribution and surface/bone volume are examples of bone geometry. These parameters can interpret bone structural stiffness or failure and can be correlated with mechanical failure. Micro-CT has become the “gold standard” for the evaluation of bone morphology and microstructure [20].

There are numerous advantages to using micro-CT in the study of bone: it allows the direct 3D measurement of bone morphology without slicing; a large volume of interest is analyzed; the analysis does not require special preparation of the sample. The assessment of bone morphology and structure is nondestructive, can be performed without removing skin and soft tissue and can be performed in vivo in anesthetized small animals. Lastly, micro-CT can provide an estimation of bone mineral density provided that appropriate standards are used.

### 1.4. Scope of the work

In this work, the effect of lipoprotein deficiency on bone quality in animal models is studied using a combination of Raman microspectroscopy and X-ray microcomputed tomography. Plasma lipoproteins are soluble protein–lipid complexes that carry out lipid transportation and metabolism in the blood. The high-density lipoprotein (HDL) class is composed of several subclasses of particles that vary in size and average molecular weight. The production of HDL requires the major cholesterol-binding protein apolipoprotein A-1 (APOA1), representing about 60% of the total protein weight in human HDL. APOA1 is a gene that, when expressed, promotes the removal of lipids from cells and prevents their accumulation [21].

In the past few years, several studies have implicated lipids and lipoprotein regulators in bone quality [21,22,23,24]. F. Pirih et al. (2012) studied the undesired effect of hyperlipidemia on bone quality. They compared wild-type (WT) and hyperlipidemic (Ldlr^−/−^) mice after placing them on a high-fat diet for 13 weeks. The repair/regeneration of the cranial bones and the mechanical properties of the femoral bones were assessed using microcomputed tomography, histological analysis, three-point bending analysis and serum analysis. The combination of the findings suggested that hyperlipidemia induces secondary hyperparathyroidism and impairs bone regeneration and mechanical strength [25]. In 2010, Tsezou A. et al. investigated the expression of genes (in particular, APOA1) regulating cholesterol efflux in human chondrocytes and osteoarthritis (OA). Cholesterol efflux gene expression was significantly lower in osteoarthritic cartilage compared to normal cartilage [26]. Blair et al. studied imbalances in the lipid metabolism of apolipoprotein-deficient mice and how they affect bone homeostasis by altering bone mass and quality. They found that bone mass was greatly reduced in deficient mice compared to their wild-type counterparts [27]. Static and dynamic histomorphometry showed that the reduced bone mass in APOA1^−/−^ mice reflected decreased bone formation. They came to the conclusion that an APOA1 deficiency generates changes in the bone-cell-precursor population that increase adipoblast and decrease osteoblast production, resulting in reduced bone mass and impaired bone quality in mice [28]. Liu et al. also stated that NHERF1-deficient mice developed osteomalacia and that their bone mechanical properties were severely degraded [28]. In the aforementioned studies, histomorphometric analysis as well as mechanical stress and molecular genetic analyses were mainly performed. Raman spectroscopy has been used for the assessment of bone quality in genetically modified organisms and has successfully identified osteoporotic bones in several studies [10,29,30,31]. Its use in organisms lacking genes or proteins, though, has not been frequent and has only recently been emergent [32]. X-ray microscopy is another nondestructive tool for the in-depth structural, morphometric and densitometric analysis of bone.

The combination of Raman microspectroscopy and X-ray microcomputed tomography is considered necessary for a more comprehensive analytical approach to bone quality assessment. Raman spectroscopy is a surface technique for studying local characteristics. Whole-bone surfaces (the cortical periosteum and endosteum, after cutting) can be scanned in a spot-like manner. Trabecular bone cannot be reached and does not offer a good surface for Raman spectra. On the other hand, microcomputed tomography provides a high-resolution three-dimensional structural image for detailed structural analysis. Raman bone metrics show compositional trends of the constituents of bone and are known to be related to its mechanical properties. Compositional data, apart from data on mineral density, cannot be assessed with micro-CT. However, a number of morphometric (geometric) parameters can be collected and also related to mechanical properties. Trabecular bone can only be assessed with micro-CT, while collagen crosslinking can only be assessed with Raman spectroscopy. In the present study, a group of animals with an apolipoprotein deficiency (“knock-out” group), which is reported to have an effect on their bone health status [27], is used in comparison with control littermates (“wild-type”). Cortical bone and trabecular bone are both studied. The composition of the two major constituents, mineral (bioapatite) and collagen, as well as their quality indices, are assessed. Bone mineral density and geometrical parameters such as bone thickness are measured. It is shown that a comprehensive examination of bone integrity requires the combination of the two imaging techniques. The conclusions are compared with mechanical data from the literature.

## 2. Results

### 2.1. Raman Spectroscopy

#### 2.1.1. Raman Spectra of Bone

**Figure 1:**
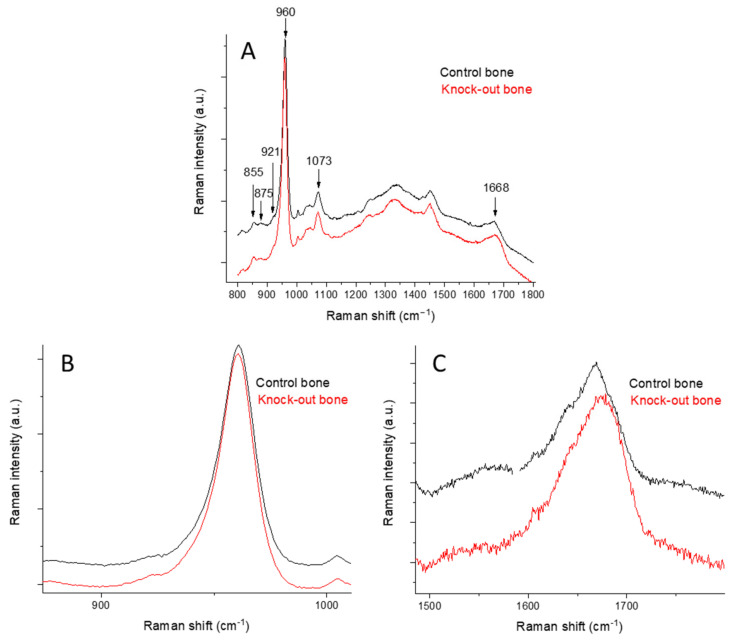
Full Raman spectra of a control femur (black line) and a knock-out femur (red line) (**A**). The peaks of interest are shown in the zoomed-in apatite region (**B**) and zoomed-in amide I region (**C**).

Raman spectra of a healthy control (wild-type) and an apolipoprotein-deficient (knock-out) femur are shown in Figure 1A. Characteristic peaks assigned to bioapatite and collagen, the major constituents, are labeled in the figure and are quoted in Table 1, and these are in accordance with the literature [15]. Figure 1B,C shows isolated and zoomed-in views of the spectral areas of apatite (Figure 1B) and amide I (Figure 1C). The regions of apatite in the two groups are identical, while a significant difference is noted in the regions of amide I.

The most widely seen mineral band is a phosphate band at 960 cm^−1^ (ν_1_PO_4_^3−^), which is characteristic of carbonated apatites. The most intense B-type carbonate band appears at 1073 cm^−1^ (ν_1_CO_3_^2−^) for bone mineral. Its exact position is sensitive to the carbonate mineral content (CO_3_^2−^) and monohydrogen phosphate (HPO_4_^2−^) content. Newly deposited mineral has a high HPO_4_^2−^ content, and the band is shifted to lower wavenumbers [33,34,35]. The full-width half-maximum of the ν_1_PO_4_^3−^ band is inversely proportional to the mineral crystallite c-axis length and is often used to assess mineral crystallinity. The strength of bone is dependent not only on the amount of mineralization but also on the degree of mineral crystallinity and the optimal distribution of different crystal sizes.

The most widely reported collagen band is amide I. This band is actually a result of several partially resolved components. The major peaks of the control and knock-out bones are at 1668 cm^−1^ and 1690 cm^−1^, and their relative intensities depend on collagen’s secondary structure. Their respective bands were initially used in infrared spectroscopy to assess the ratio of the trivalent nonreducible crosslink pyridinoline to divalent (immature) reducible collagen crosslink dihydroxylysinonorleucine [36,37]. These ratio changes have been used in Raman spectroscopy to infer the presence of pathological situations.

#### 2.1.2. Raman Study of Femoral and Tibial Cortical Bones

Raman microspectra were collected from various spots on the femur and tibia bone periosteum in the mid-diaphysis region for the two animal groups. Since this type of information comes exclusively from the surface, endosteum on the transverse section of the femurs were studied. The results for femurs and tibiae were similar, and they were grouped together.

##### Mineral-to-Matrix Ratio

**Figure 2:**
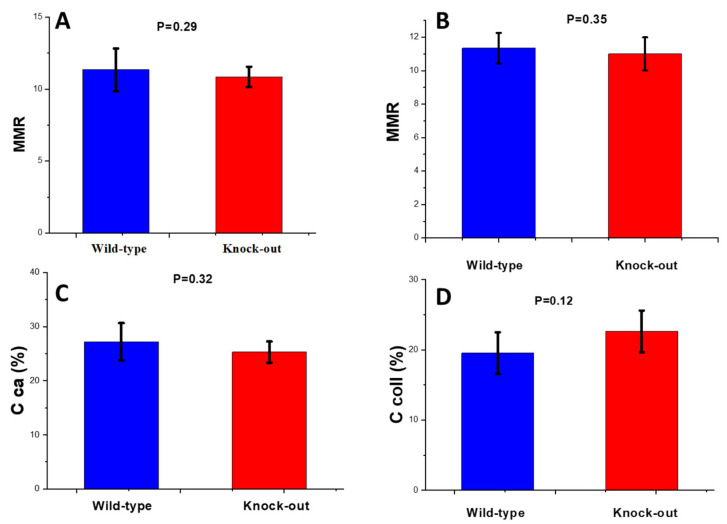
Mineral-to-matrix Raman band ratio for wild-type and knock-out (**A**) periosteum and (**B**) endosteum of mice; (**C**) average calcium content of WT and knock-out mice using AAS and (**D**) average collagen content of WT and knock-out mice using TGA. Data are presented as mean ± S.D.

**Figure 3:**
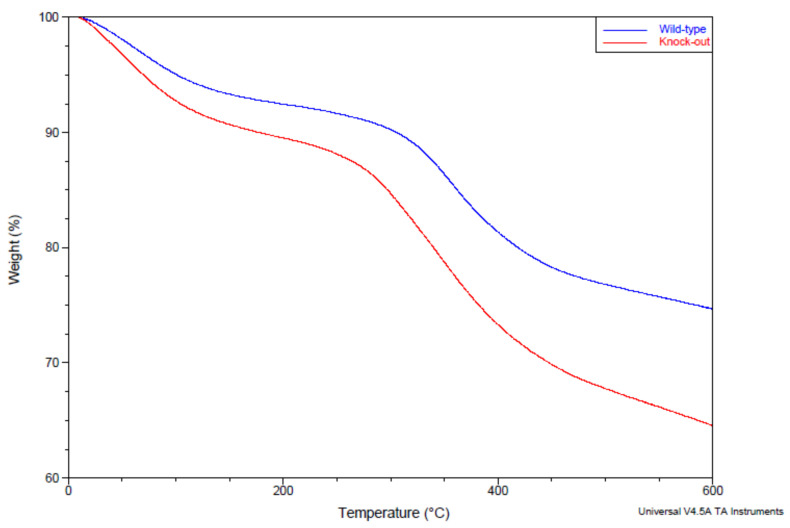
Typical TGA thermographs for control and wild-type groups.

No significant difference was observed in the MMR between the two animal groups for both periosteum (Figure 2A) and endosteum (Figure 2B). This MMR stability implies that mineralization remained stable between the two groups. To confirm this, measurements of calcium in the mineral content were performed via atomic absorption spectrometry (AAS). No significant difference was observed between the two animal groups (Figure 2C). For collagen quantity analysis, thermal gravimetric analysis (TGA) was performed. Typical thermographs for the wild-type and knock-out groups are shown in Figure 3. A slight increase in the collagen content in the knock-out mice was observed in the TGA curves. However, as shown in Figure 2D, no statistically significant change was recorded between the wild-type and knock-out groups. It should be noted that the AAS and TGA results are cumulative for periosteum and endosteum.

##### Carbonate-to-Phosphate Ratio (CPR)

**Figure 4:**
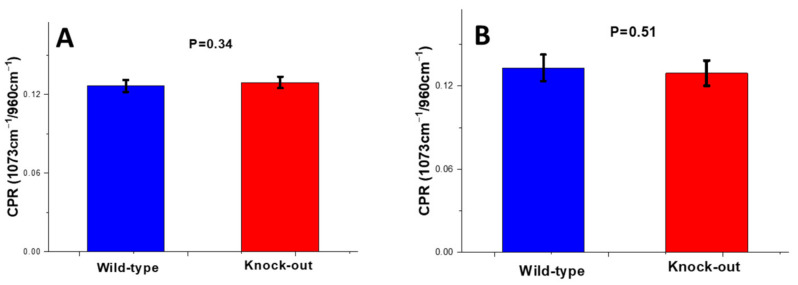
Carbonate-to-phosphate Raman band ratio for wild-type and knock-out (**A**) periosteum and (**B**) endosteum of mice. Data are presented as mean ± S.D.

After deconvolution of the carbonate band (1073 cm^−1^), the CPR ratio of each bone was calculated using Equation (2). No significant change in the carbonate content of the cortical and trabecular bone was observed between the wild-type and knock-out groups (Figure 4).

##### Crystallinity

For the assessment of mineral crystallinity, the inverse of the full-width half-maximum of 960 cm^−1^ (1/FWHM (960 cm^−1^)) was calculated.

**Figure 5:**
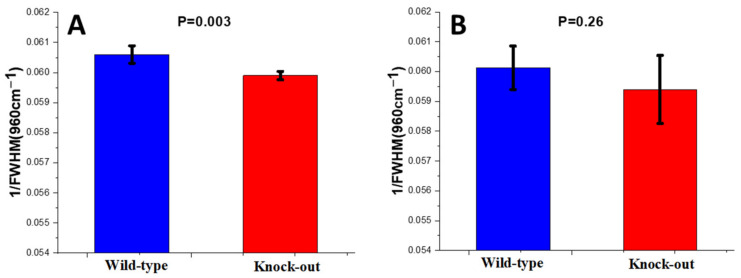
Crystallinity of wild-type and knock-out (**A**) periosteum and (**B**) endosteum of mice. Data are presented as mean ± S.D.

The crystallinity of bioapatite was found to be decreased in the knock-out mice compared to the wild-type mice in the periosteum of their bones, while it remained stable in their endosteum (Figure 5).

##### Crosslinking Ratio (CLR)

**Figure 6:**
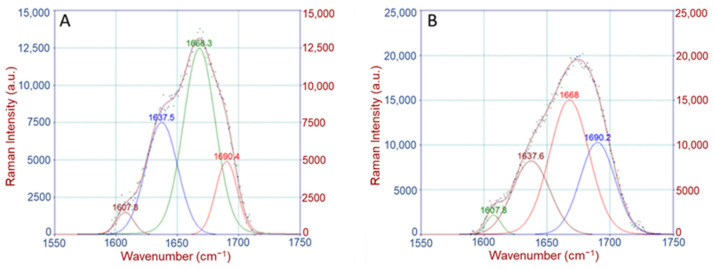
Deconvolution and band fitting under the amide I area for (**A**) wild-type and (**B**) knock-out femurs.

The organic content of the bones was characterized by the region of 1550–1750 cm^−1^. The peak at 1668 cm^−1^ is assigned to the C=O stretch of the nonreducible (trivalent) crosslinks of collagen (amide I) and that at 1690 cm^−1^ corresponds to the respective reducible (divalent) crosslinks. A change in the shape of the amide I envelope was observed in the knock-out mice compared to the wild-type mice for both periosteum and endosteum. Further analysis showed that this resulted from a change in the ratio of the sub-bands lying under the amide I area (Figure 6).

**Figure 7:**
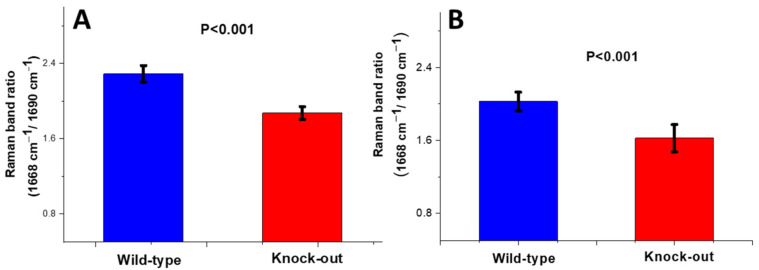
Crosslinking Raman band ratio for (**A**) periosteum and (**B**) endosteum of mice. Data are presented as mean ± S.D.

Calculation of the band area ratio (1668 cm^−1^/1690 cm^−1^) revealed a substantial decrease (approx. 20%) in the knock-out mice compared to the wild type for both periosteum and endosteum (Figure 7).

### 2.2. X-ray Microcomputed Tomography

#### 2.2.1. Density and Morphometric Parameters of the Cortical Bones

**Figure 8:**
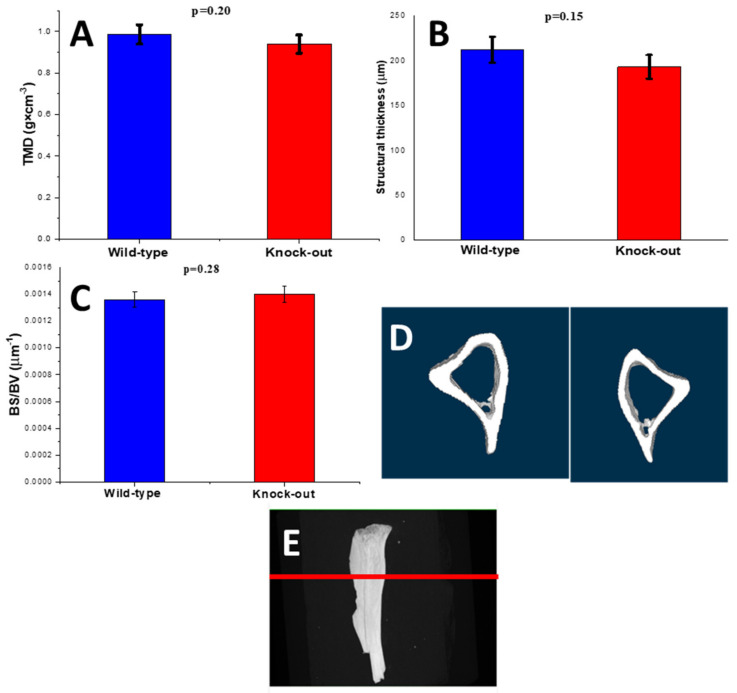
(**A**) Tissue mineral density (TMD), (**B**) average structural thickness (St.Th), (**C**) bone surface-to-volume ratio (BS/BV), (**D**) 3D microcomputed tomography pictures of wild-type (**left**) and knock-out (**right**) tibia cortical bones and (**E**) 3D microcomputed tomography pictures of the position of the tibia slices shown in (**D**).

The tissue mineral density (TMD) of the knock-out cortical bones exhibited no variation compared to the TMD of the wild-type bones (*p* = 0.20; Figure 8A). The structural thickness (St.Th) did not vary between them either (Figure 8B). The bone surface-to-volume ratio (BS/BV) remained stable as well (Figure 8C). It appears that no significant density or morphometric change was noted for the two groups. Three-dimensional micro-CT images illustrate these findings (Figure 8D).

#### 2.2.2. Density and Morphometric Parameters of the Trabecular Bones

The trabecular bones were assessed using the BMD for tissue density as well as using the TMD for the density of the trabecular material itself.

**Figure 9:**
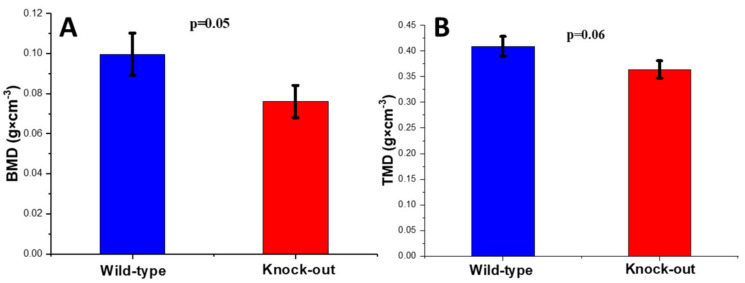
(**A**) Bone mineral density (BMD) and (**B**) tissue mineral density (TMD) of wild-type and knock-out tibia trabecular bones.

A completely different state was noted for the trabecular bones in the two groups. The bone mineral density (BMD) of the knock-out trabecular bones was (statistically) lower than the respective value for the wild-type bones. The BMD exhibited a reduction of 23.7%, suggesting a severe reduction in the bone mass (Figure 9A). Further to this, the TMD of the knock-out bones, which represents the density of the bones as a material, also exhibited a reduction, though not a statistically significant one, of 11.1% compared to the wild-type bones (Figure 9B).

**Figure 10:**
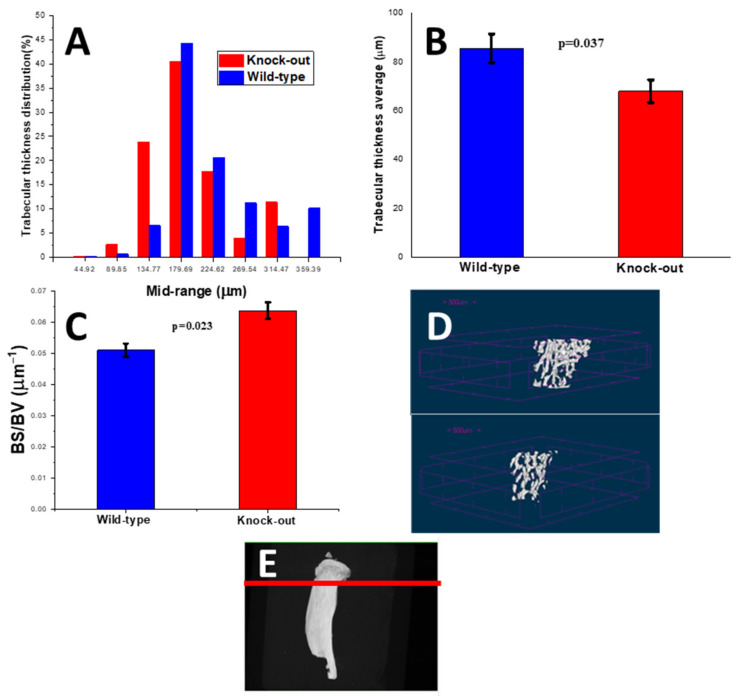
(**A**) Trabecular thickness (Tb.Th) distributions, (**B**) average trabecular thickness, (**C**) bone surface-to-volume ratio (BS/BV), (**D**) 3D microcomputed tomography pictures of wild-type (**top**) and knock-out (**bottom**) trabecular bones for wild-type and knock-out tibiae and (**E**) 3D microcomputed tomography pictures of the position of the tibia slices shown in (**D**).

The distribution of thickness in the knock-out animal models shifted to lower values compared to those of the wild-type models (Figure 10A). A decrease of 20.5% in the average thickness value (Tb.Th) was also recorded (Figure 10B). The 3D micro-CT images of the two groups (Figure 10D) illustrate these results.

The bone surface-to-volume ratio (BS/BV) was notably increased (24.8%; statistically significant) (Figure 10C).

## 3. Discussion

In the trabecular part of the apolipoprotein-deficient tibiae, a reduction (23.7%) in bone mineral density (BMD) was observed in comparison with control littermates. This suggested a (statistically) significant reduction in bone mass; for the same volume, the quantity of bone was lower. This was further confirmed by three-dimensional micro-CT images, which showed that the trabecular network was sparser, although it did not lose its spongy architecture. The trabecular thickness of the knock-out bones was severely reduced (20.5% was the average value) compared to the control, while the thickness distribution of the knock-out animals shifted towards lower values.

The bone surface-to-volume ratio of the trabeculae notably increased (24.8%). The surface of the knock-out bones available for degradation increased.

All these findings converged to the conclusion of severe osteoporosis [38,39]. Blair et al. [27] also reported a reduced BMD in the trabecular bones of the lumbar vertebrae of apolipoprotein-deficient mice. Osteoporotic trabecular bones are expected to exhibit degraded mechanical behavior and are expected to be prone to fracturing [38].

For femoral and tibial cortical bones, the mineral-to-matrix Raman ratio (MMR) of the knock-out bones was found to be statistically similar to that of the wild-type counterparts (Figure 2A). This was valid throughout the bone volume. The mineral quantity, as measured by atomic absorption spectrometry, was also identical. Carbonate substitution, which is related to bone friability [40], also did not change. Additionally, the TMD and all the morphometric parameters (structural thickness and bone surface-to-volume ratio) were similar. The only characteristic to contradict this was the crystallinity of the knock-out periosteum (11.6% reduction relative to the control). Knock-out mineral crystallinity was lower compared to the wild-type value. Such a reduction has been said in the literature to relate to degraded mechanical strength [38].

As far as the collagen quantity is concerned, which was measured via thermogravimetric analysis, this remained stable between the two animal groups. However, crosslinking (nonreducible trivalent/reducible divalent crosslinks), which was evaluated by Raman spectroscopy (CLR), differed between the groups to a significant degree throughout the cross-section of the bones (periosteum and endosteum), with the knock-out bones having the lowest value in all the cases (periosteum and endosteum). It is worth noting that the percentage reductions were also similar: 18.2% for the periosteum in the knock-out bones and 19.8% in the knock-out endosteume. This suggests that there was a decrease in the trivalent (mature) crosslinks (pyridinolines (HP and LP) and pyrroles) with regard to the divalent crosslinks. Since the divalent crosslinks are the first to form, and since they are reduced to trivalent crosslinks during a collagen network’s maturation [10], it was concluded that the knock-out network’s maturity was lower compared to the wild-type network’s maturity, although the collagen quantity was the same between the two groups. It is also worth noting that this was true throughout the femoral and tibial periosteum and endosteum at approximately the same percentage (≈19%).

Blair et al. reported that they found a reduced fracture load (the energy required for crack initiation was reduced, so the bones cracked more easily) and a large reduction (60%) in elasticity (the stress required to break the bones was reduced, so the bones became stiffer) in apolipoprotein-deficient mice [27].

Liu et al. also stated that the Raman crosslink ratio reduced by almost 32% in NHERF-deficient mice compared to the control. The bone mass remained stable between the wild-type and knock-out mice. The fracture force (the energy required for crack initiation) was reduced by approximately 25% [28].

EMB McNerny and their coworkers examined the impact of lysyl oxidase (necessary for crosslinking) inhibition on the crosslink profile and mechanical properties [10]. They found reduced (pyridinolines) trivalent crosslinks compared with the controls, but no differences in the immature crosslinks (DHLNL and HLNL or pentosidine) were detected. The crosslink maturity ratios were significantly reduced. A loss in bone strength and a decrease in fracture toughness were recorded and correlated with mature crosslink reduction. The mineral measures were not affected by the specific treatment. The TMD was also not found to be a significant factor in explaining the mechanical properties.

On the other hand, exercise was found to increase the number of total mature crosslinks as well as the relative crosslink maturity, which had a positive impact on mechanical properties [9]. The recovery effect of exercise was not explained by greater mineralization, and it did not increase the TMD. It was concluded that the mineral content controls rigidity and strength and that the organic matrix provides bone ductility and toughness.

In their work, Morris et al. stated that the mineral-to-matrix ratio and tissue mineral density should not be related to mechanical properties, such as bone hardness, and that crosslinking should. Bone mineral density has traditionally been clinically used to evaluate bone mass as a surrogate for bone strength. However, BMD does not always predict fracture risk, and it is now well accepted that both bone quality and bone mass are important factors in determining bone strength. Bone quality is interconnected with biomechanical properties [2,29].

Resistance to fracturing is dependent not only on the quantity and distribution of bone but also on the quality of the bone material. The toughness of bone stems largely from the properties of its polymeric organic matrix. Covalent crosslinks are responsible for network stabilization. Trivalent crosslinks play a greater role than divalent crosslinks in stabilizing the organic matrix of bone.

## 4. Materials and Methods

### 4.1. Mice

Twelve-week-old apolipoprotein-deficient (knock-out, KO) male mice, which were backcrossed for C57BL/6 10 generations, and control C57BL/6 (wild-type, WT) mice (Jackson Labs, Bar Harbor, ME, USA) were maintained on a standard chow diet, containing 29% protein, 60% carbohydrates and 11% fat (Mucedola SRL, Milan, Italy), ad libitum in a 12 h dark/light cycle (light from 0700 h to 1900 h). Animals were genotyped via tail-DNA PCR. Groups of mice had similar average body weight, plasma cholesterol, triglycerides and glucose. Mice were caged individually. At 12 weeks, the mice were killed. Femurs and tibiae were collected for analysis in accordance with published standards [27].

Right femurs and tibiae from five wild-type and six apolipoprotein-deficient animals were used. Soft tissue was removed by means of a scalpel. Bone samples were then wrapped in a gauze, which was previously immersed in phosphate-buffered saline (PBS), and were maintained in a freezer (−20 °C) until use. Before analysis, the samples were left to thaw to room temperature.

### 4.2. Raman Spectroscopy

A Raman spectrometer (InVia Reflex Raman spectrometer; Renishaw, New Mills Wotton-under-Edge Gloucestershire, UK) equipped with an optical microscope (research-grade Leica DMLM microscope, Leica Microsystems, Wetzlar, Germany) and a laser with a 785 nm excitation line was used. The laser line was focused through a 20x objective lens on the sample. The system was equipped with a CCD detector (Peltier cooled and near-infrared enhanced). The power of the incident laser was 250 mW. The laser exposure time was 10 s; the laser power was set at 80%; there were 10 accumulations for all measurements.

Raman spectra were collected from different spots on femur and tibia periosteum in the entire diaphysis region. Next, the femurs were cut in the mid-diaphysis region by means of a scalpel. Raman spectra were also recorded from the endosteum.

Four regions of interest were isolated for further analysis in the Raman spectra: 800–900 cm^−1^ (prolines), 900–990 cm^−1^ (apatite), 990–1140 cm^−1^ (carbonate) and the amide I envelope at 1590–1730 cm^−1^. All regions in the Raman spectra were isolated and baselined. Further analysis included deconvolution and curve fitting of the sub-bands using Peakfit software (Peakfit© v4.0; Jandel Scientific, San Rafael, CA, USA). The typical spectral resolution was 2 cm^−1^. Instrument response (laser power and wavenumber) was checked by recording the spectrum of Si.

#### 4.2.1. Raman Metrics

Raman metrics, calculated as band-intensity ratios, included the mineral-to-matrix ratio (MMR, Equation (1)), carbonate-to-phosphate ratio (CPR, Equation (2)) and collagen crosslink (or collagen maturity) ratio (CLR, Equation (3)). Crystallinity was calculated as the inverse of the phosphate 960 cm^−1^ band width.

MMR = {I(960 cm^−1^)/[I(855 cm^−1^) + I(875 cm^−1^) + I(921 cm^−1^)]}, (1)

CPR = I(1073 cm^−1^)/I(960 cm^−1^) (2)

CLR = I(1668 cm^−1^)/I(1690 cm^−1^) (3)

#### 4.2.2. Raman Band Deconvolution

**Figure 11:**
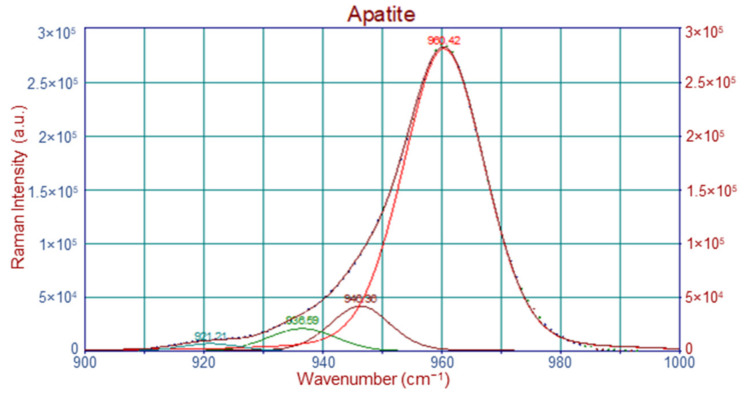
Deconvolution and band fitting under the apatite area for a healthy femur.

Spectral processing included baselining, deconvolution and curve fitting of the sub-bands [1]. An example is shown in Figure 11. The peaks at 921.21, 936.59 and 946.36 cm^−1^ lie laterally under the major phosphate peak at 960.42 cm^−1^.

The band ratios of MMR, CPR, CLR and crystallinity, corresponding to femurs and tibiae of the same group (wild-type or knock-out group), were pooled together. Average data from different groups were compared. The results are stated as average values ± standard deviation, and a *t*-test was used for statistical evaluation. In all tests, *p* < 0.05 was considered significant.

### 4.3. Atomic Absorption Spectroscopy

Next, atomic absorption force spectrometry was used for the calculation of Ca in mice femurs. A AA300 atomic absorption spectrophotometer equipped with an HGA800 graphite furnace (PerkinElmer LAS Chalfont Road, Seer Green, Beaconsfield, UK) was used. Carefully weighed portions of all femurs were suspended in HNO_3_ solutions to achieve the complete dilution of bioapatite. After filtration, dispersions of low concentrations were prepared. The standard addition method was employed for quantitative analysis.

### 4.4. Thermal Gravimetric Analysis

A TGA Q50 V5.3 Build 171 was used for thermal analysis. Amounts (5–10 mg) of all femurs were placed in the TGA furnace, and their thermographs were recorded. The region of temperature was 25–800 °C, and the ramp was 15 °C/min.

### 4.5. Microcomputed Tomography

The specimens’ microarchitecture was examined via microcomputed tomography (micro-CT). Tibiae were analyzed here, as femurs were destroyed during AAS and TGA analysis. Each tibia bone sample was scanned using a Bruker SkyScan 1174 (Bruker Belgium SA, Kontich, Belgium) micro-CT scanner operated at 50 kV and 800 μA. The parameters for each measurement were a 0.5 mm Al filter, image pixel size set at 11.23 μm, 0.500-degree rotation step and frame averaging of 5, while the rotation range was set at 0° to 180° for all samples. After acquisition, the images were reconstructed (NRecon, Bruker Belgium SA, Kontich, Belgium) in order to apply noise-reduction and beam-hardening corrections to the data (beam-hardening correction of 36, ring-artifact correction of 8 and image-smoothing correction of 2). To ensure apparatus stability and to provide a calibration standard for BMD (bone density) calculations, reference phantoms (Bruker Belgium SA, Kontich, Belgium) were employed. These were two cylindrical rods consisting of epoxy resin and containing fine hydroxyapatite powder (CaHAp) in known concentrations of 0.25 and 0.75 g/cm^3^. Calibration of the system was performed using the same scanning and reconstruction parameters that were chosen for the tibia samples. Care was taken to ensure appropriate adjustment of the contrast range of the images so as to achieve attenuation coefficient limits suitable for both the calibration phantoms and the various samples. With that in mind, the selected values were an attenuation coefficient minimum of 0.00 and an attenuation coefficient maximum of 0.11 cm^2^/g.

Distinction of the cortical bone from the trabecular bone was carried out by manually drawing the corresponding areas of interest. For that to be consistently achieved, the growth plate was selected each time as a reference slice, and a specific offset and height were set separately for cortical (measured at the diaphysis) and trabecular (measured at the metaphysis) measurements.

In order to evaluate a bone specimen and to use the resulting numeral values, binarization of the dataset was carried out. During binary selection, two thresholds were chosen (upper and lower) within an 8-bit gray scale, which varied from 0 to 255. The upper and lower thresholds were selected so as to optimally differentiate between bone and nonbone material. Different binarization thresholds were selected for cortical bone and the trabecular bone, namely, 100–255 for the cortical bones and 55–255 for the trabecular bones [41].

Finally, the average values of the following densitometric and morphometric parameters and the distribution of values were calculated (CTAn, Bruker Belgium SA, Kontich, Belgium): bone mineral density (BMD) for trabecular bone, tissue mineral density (TMD) for both cortical and trabecular bones, bone surface-to-volume ratio (BS/BV), structural thickness (St.Th) for cortical and trabecular bone and thickness (Tb.Th) for trabecular bone.

## 5. Conclusions

This study revealed a severe reduction in the bone mineral density of the trabecular bone of apolipoprotein-deficient mice. Cortical femurs and tibiae of the control and knock-out groups appeared to be equally mineralized. However, the collagen network crosslinking ratio for the knock-out cortical bones was shifted to lower values, which was ascribed to a decrease in trivalent crosslinks compared with divalent crosslinks. This reduction is related, according to the literature, to reduced fracture resistance. These findings reveal that the knock-out bone was osteoporotic.

All of the data came from a combination of Raman microspectroscopy and X-ray microcomputed tomography. Although an accurate depiction of bone quality was given, no information could be given on the exact way this protein affects the quality of bone, leading to osteoporosis.

## Figures and Tables

**Table 1:** Characteristic peaks in the Raman spectra of control and knock-out femora and their assignment to bond vibrations of their constituents.

Raman Shift (cm^−1^)	Band Assignment
855	ν(C-C), collagen proline
875	ν(C-C), collagen hydroxyproline
921	ν(C-C), collagen proline
937	ν(C-C), proline and protein backbone
946	ν_1_PO_4_^3−^
960	ν_1_PO_4_^3−^, bone mineral
1003	ν(C-C), phenylalanine
1035	ν_3_PO_4_^3−^
1050	ν_3_PO_4_^3−^
1060	proteoglycan, lipids, collagen, ν_3_PO_4_^3−^
1073	ν_1_CO_3_^2−^
1087	ν_3_PO_4_^3−^, ν_1_CO_3_^2−^
1637	ν(C=C)
1668	amide I, ν(C=O)
1690	amide I, β-sheets, disordered secondary structure

## Data Availability

Data are not available.

## Sample Availability

Not available.
